# Vacuum mattress or long spine board: which method of spinal stabilisation in trauma patients is more time consuming? A simulation study

**DOI:** 10.1186/s13049-021-00854-w

**Published:** 2021-03-11

**Authors:** Roessler MS, M Riffelmann, N Kunze-Szikszay, M Lier, O Schmid, H Haus, S Schneider, Heuer JF

**Affiliations:** 1 Department for Anaesthesiology, University Medical Center Göttingen, Robert-Koch-Strasse 40, 37075 Göttingen, Germany; 2 Praxis Schmallenberg, Obringhauser Strasse 4, 57392 Schmallenberg, Germany; 3 Department of Anaesthesiology, Intensive Care and Pain Medicine, Eichsfeld Clinic, Windische Gasse 112, 37308 Heilbad Heiligenstadt, Germany; 4 Department of Medical Statistics, University Medical Center Göttingen, Robert-Koch-Strasse 40, 37075 Göttingen, Germany; 5 Department of Anaesthesiology, Intensive-Care-, Emergency- and Pain-Medicine, Augusta Krankenanstalt Bochum, Bergstrasse 26, 44791 Bochum, Germany

**Keywords:** Long spine board, Prehospital trauma treatment, Spinal stabilisation, Vacuum mattress

## Abstract

**Background:**

Spinal stabilisation is recommended for prehospital trauma treatment. In Germany, vacuum mattresses are traditionally used for spinal stabilisation, whereas in anglo-american countries, long spine boards are preferred. While it is recommended that the on-scene time is as short as possible, even less than 10 minutes for unstable patients, spinal stabilisation is a time-consuming procedure. For this reason, the time needed for spinal stabilisation may prevent the on-scene time from being brief. The aim of this simulation study was to compare the time required for spinal stabilisation between a scoop stretcher in conjunction with a vacuum mattress and a long spine board.

**Methods:**

Medical personnel of different professions were asked to perform spinal immobilizations with both methods. A total of 172 volunteers were immobilized under ideal conditions as well as under realistic conditions. A vacuum mattress was used for 78 spinal stabilisations, and a long spinal board was used for 94. The duration of the procedures were measured by video analysis.

**Results:**

Under ideal conditions, spinal stabilisation on a vacuum mattress and a spine board required 254.4 s (95 % CI 235.6–273.2 s) and 83.4 s (95 % CI 77.5–89.3 s), respectively (*p* < 0.01). Under realistic conditions, the vacuum mattress and spine board required 358.3 s (95 % CI 316.0–400.6 s) and 112.6 s (95 % CI 102.6–122.6 s), respectively (*p* < 0.01).

**Conclusions:**

Spinal stabilisation for trauma patients is significantly more time consuming on a vacuum mattress than on a long spine board. Considering that the prehospital time of EMS should not exceed 60 minutes and the on-scene time should not exceed 30 minutes or even 10 minutes if the patient is in extremis, based on our results, spinal stabilisation on a vacuum mattress may consume more than 20 % of the recommended on-scene time. In contrast, stabilisation on a spine board requires only one third of the time required for that on a vacuum mattress.

We conclude that a long spine board may be feasible for spinal stabilisation for critical trauma patients with timesensitive life threatening ABCDE-problems to ensure the shortest possible on-scene time for prehospital trauma treatment, not least if a patient has to be rescued from an open or inaccessible terrain, especially that with uneven overgrown land.

## Background

Spinal stabilisation (SS) is one of the standard procedures performed for prehospital trauma treatment [1] and is performed for the majority of patients with severe injuries caused by blunt trauma [2]. The aim of SS is to minimize the risk of secondary spinal cord damage in suspected injuries to the spinal column and is recommended in trauma patients with potential spinal injury [3]. Furthermore, SS helps reduce blood loss and pain when fractures of the pelvis and/or long bones are suspected [4].

A weak recommendation has been made that ABCDE-stable trauma patients with risk of a secondary spinal cord injury should undergo spinal stabilisation on a vacuum mattress instead on a hard backboard. This recommendation is based on the possible development of discomfort, pain and pressure ulcers as well as on the questionable efficacy according restriction of lateral movement if patients are transported on a hard backboard [5]. But the evidence for this recommendation is very low mostly due to the fact that the data were extrapolated from either cadaver studies or studies with healthy volunteers [6].

Nevertheless, no randomized controlled trials have compared the effects of different methods of SS with regard to mortality, neurological disability and spinal stability in trauma patients [7].

However, stabilisation is a relatively time-consuming procedure, and no other measures can be executed during the stabilisation procedure. This should be realized since time is of great importance in prehospital trauma treatment, and the on-scene time is considered an indicator of process quality. Medical associations recommend that the period from when the emergency call is received to when the patient is admitted to the hospital should not exceed 60 minutes because it is suspected that longer prehospital treatment times of trauma patients are associated with higher morbidity and mortality rates even though this remains controversial [1, 8–11]. After taking into consideration the response time of emergency medical services as well as the time needed for transport from the scene to a hospital, the on-scene time should not exceed more than 30 minutes. In severely injured patients with uncontrollable haemorrhage – which is frequently seen in penetrating trauma due to stab or gun shot wounds - the recommended on-scene time is even less than 10 minutes and is referred to as “the platinum ten” [8, 12]. While SS is not recommended to be routinely used in penetrating trauma as it may be more harm than good [13] it must be recognized that spinal injuries sustained by blasts may occur in the battlefield as well as in terrorist attacks [14].

The established methods and devices used for SS in the prehospital stetting are the placement of a patient either on a long spine board (LSB) [2] or on a vacuum mattress (VM) in conjunction with a rigid collar or head blocks. In anglo-american countries, the LSB is the gold standard. In Europe, especially in Germany, the VM is widely used, and it is part of the standardized equipment in type B/C ambulances [15].

Since time is of great importance in the prehospital phase, the method used for SS may greatly affect the on-scene time. Thus, in this study, whether the use of a VM or an LSB is superior regarding the time required for SS was investigated.

## Methods

### Study design and data collection

**Fig. 1:**
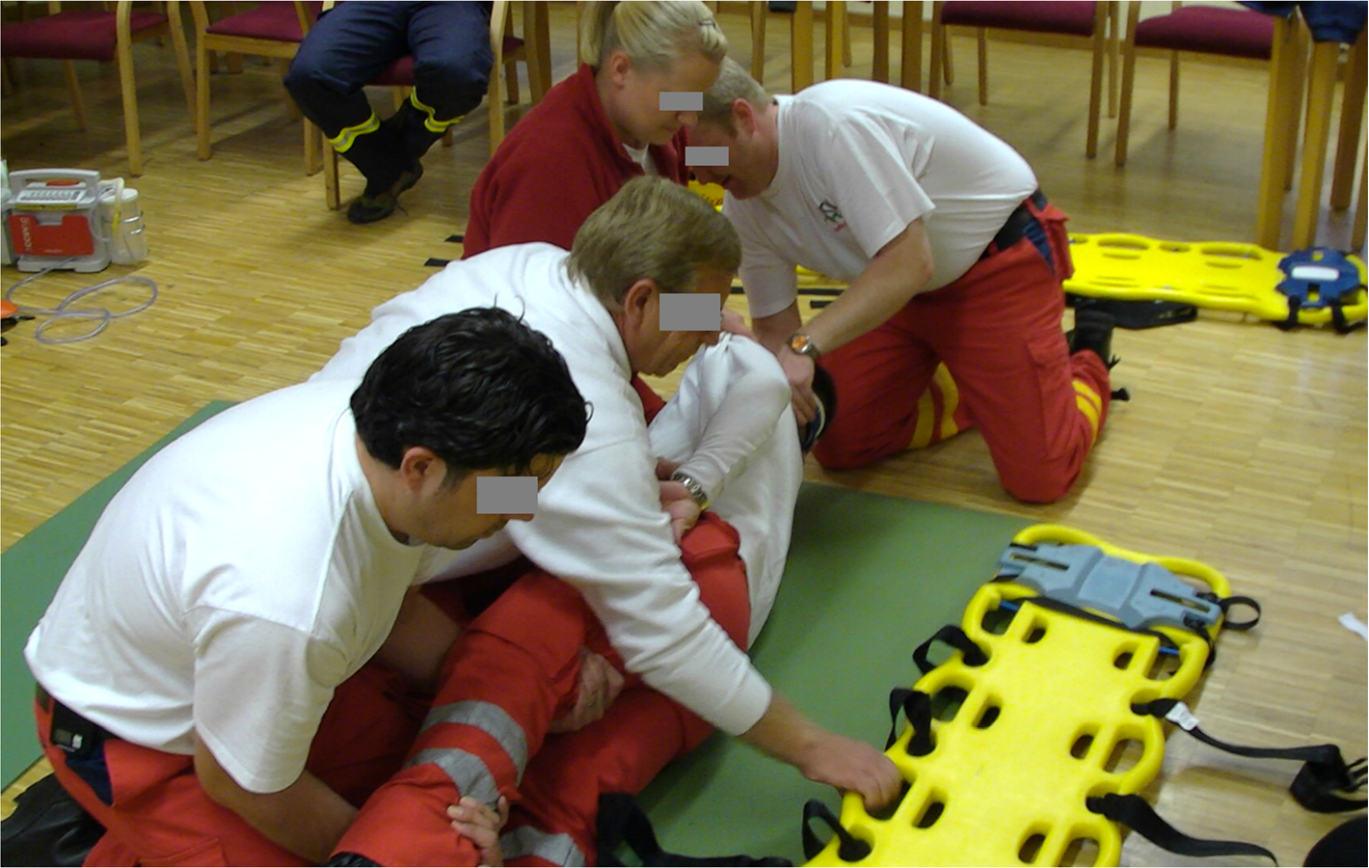
ideal conditions stabilisation on an LSB

**Fig. 2:**
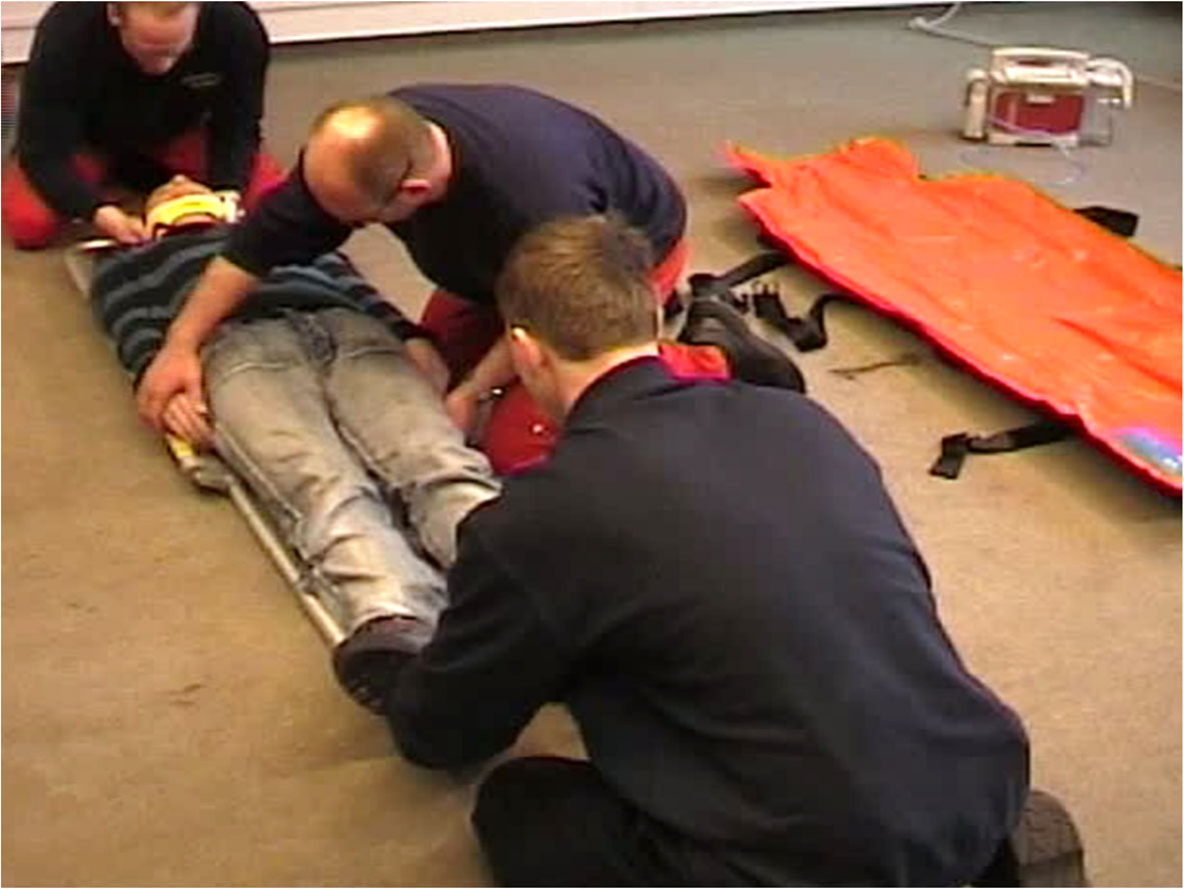
ideal conditions stabilisation on a VM (positioning on a scoop stretcher)

**Fig. 3:**
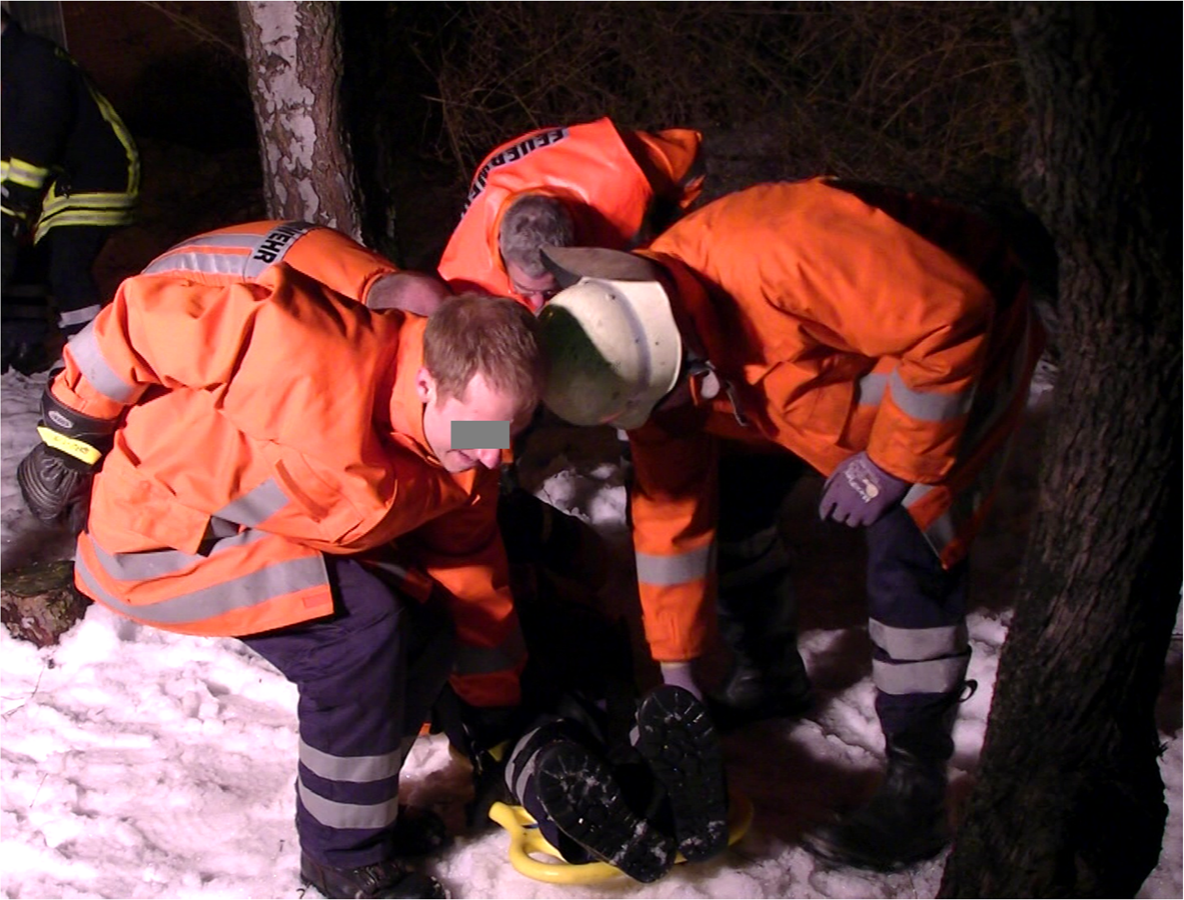
realistic conditions stabilisation on an LSB

**Fig. 4:**
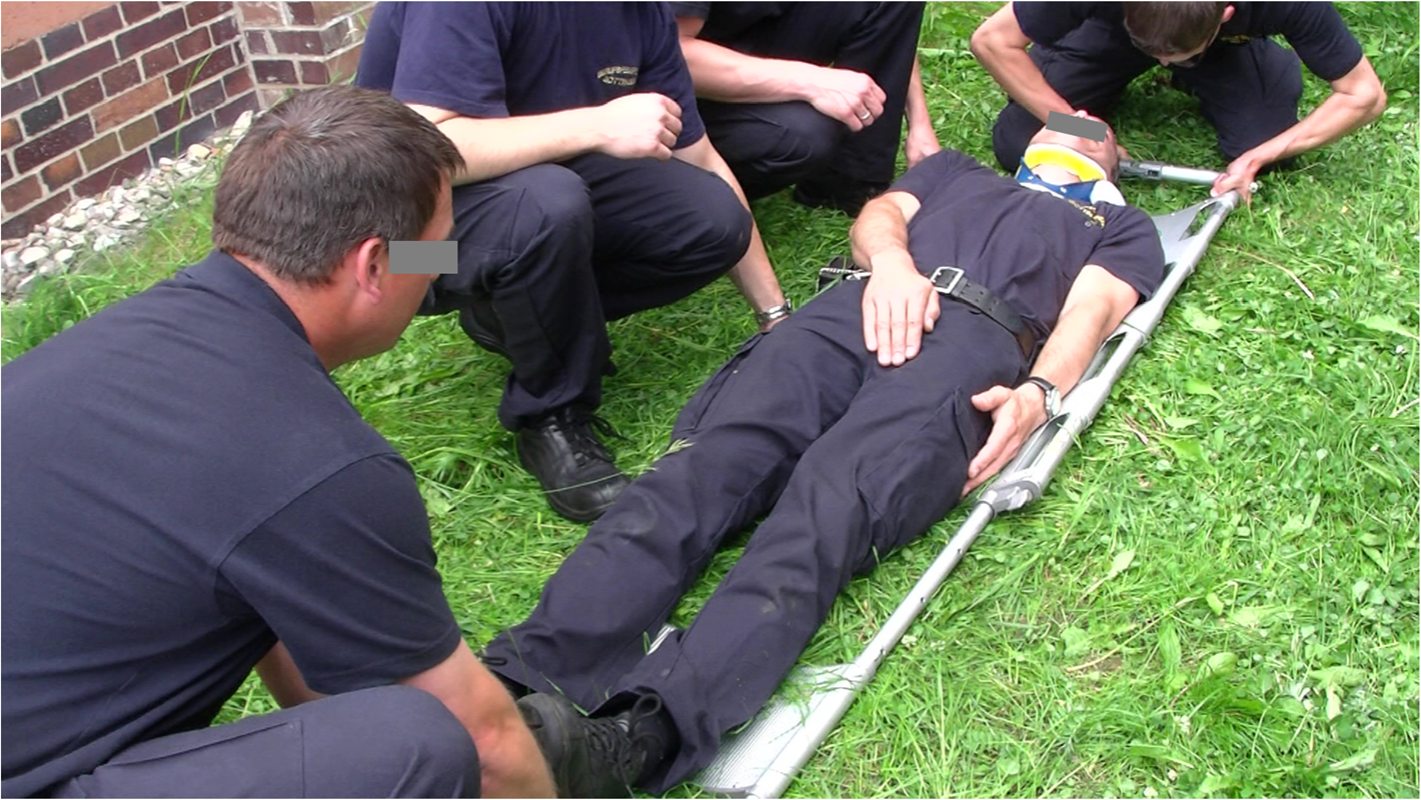
realistic conditions stabilisation on a VM (positioning on a scoop stretcher)

This study has been approved by the ethics committee of the Georg-August-University of Goettingen and included both clinical and experimental research in a two-tier model to evaluate and compare the time needed for SS in healthy participants who had to be placed either on an LSB or on a VM. Stabilisation was carried out both in ideal conditions (indoors, level ground) (see Figs. 1 and 2) and in realistic conditions (outdoors, overgrown land, open farmland) (see Figs. 3 and 4) in a standardized manner.

The same, brand-new stabilisation devices were used throughout the study. The LSB used was the BaXstrap spineboard together with the SpeedBlocks™ head immobilization system (Laerdal Medical GmbH, Pucheim, Germany). The VM used was a multiple-chamber vacuum mattress, model 814 K (Schnitzler Rettungsprodukte GmbH & Co., KG, Niederkassel-Mondorf, Germany). A vacuum was generated using an ACCUVAC Rescue suction pump (WEINMANN Emergency Technology Gmbh & Co., KG, Hamburg, Germany). A scoop stretcher, art. no. 0601035 (W. Söhngen Gmbh Erste Hilfe Notfallmedizin; Taunusstein-Wehen, Germany), was utilized to lift and position the participant on the VM.

A rigid collar, Stifneck®Select™ (Laerdal Medical GmbH, Pucheim, Germany), was applied on all volunteers prior to the stabilisation manoeuvre so that this procedure did not influence the time needed for the SS manoeuvre itself.

**Fig. 5:**
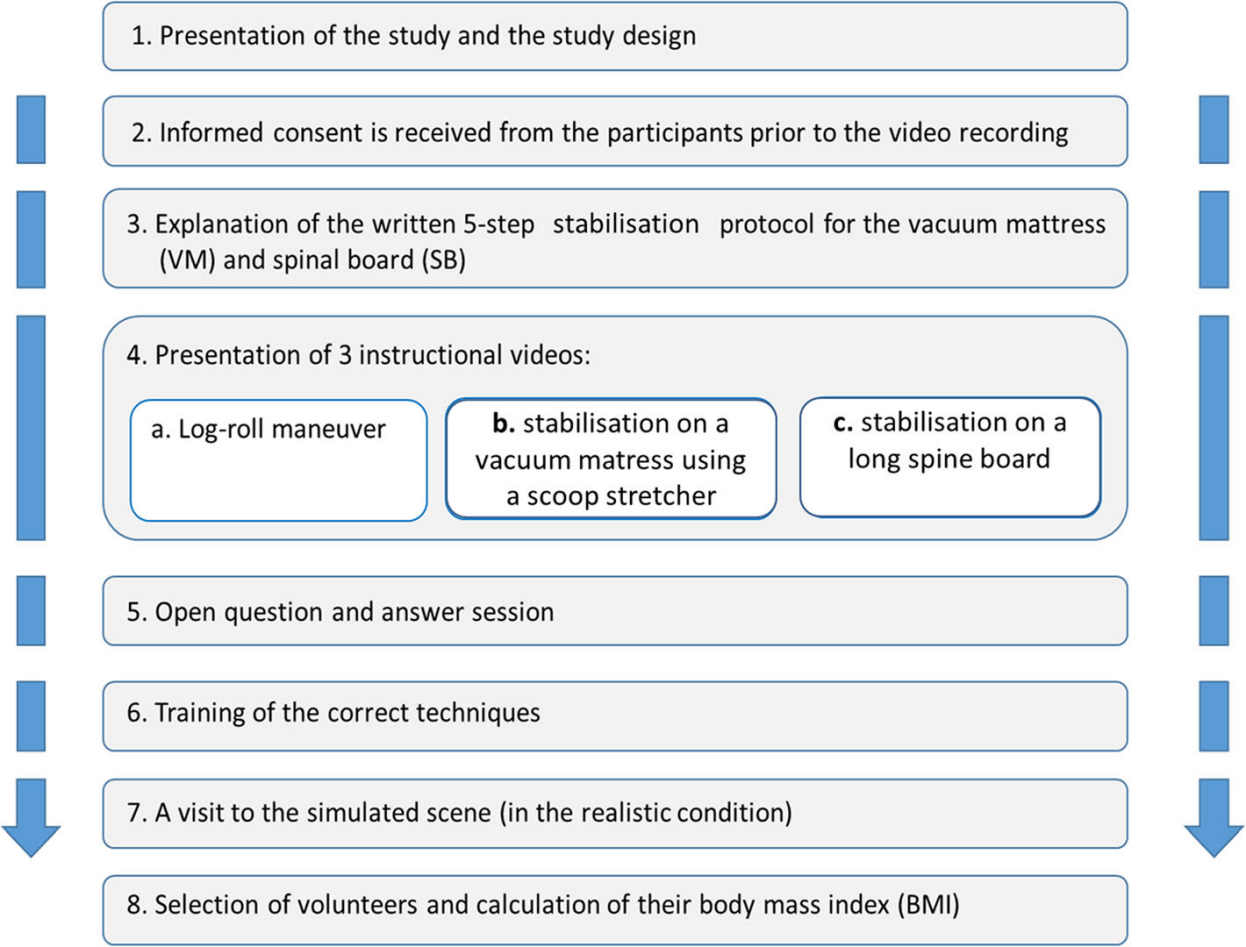
Study protocol

All the candidates performing an SS manoeuvre were thoroughly instructed to execute the following study protocol (See Fig. 5).

Stabilisation was executed by a team of four persons since four persons are usually present on the scene in the German two-tier system, where an ambulance – staffed with two paramedics – and either an emergency physician´s vehicle or a rescue helicopter – staffed with a paramedic and an emergency physician – arrive at almost the same time on the scene if there is a suspicion that the patient is severely injured. The team was made up of one team leader, positioned at the head of the patient, and three team members who were positioned alongside the patient. The team called into action consisted of either medical students in their last year, physicians participating in a board certified course for prehospital emergency medicine, paramedics, paramedics in training or firefighters.

The spinal stabilisation procedures were executed as follows:

The team leader was responsible for the manual in line stabilisation of the cervical spine and was in command for the execution of the log roll manoeuvre. The team members had to place their hands on the opposite site of the patient´s body: one on the shoulder and iliac crest, one on waist and below the knee and one below the distal femur and below the ankle. On command the manoeuvre was executed.

For stabilisation on a VM the rotation angle had to be about 15 degrees so that one of the two parts of the scoop stretcher could be placed below. Afterwards the team members changed position to the other side of the patient and repeated the manoeuvre. Both parts of the scoop stretcher were now assembled and the patient was lifted over onto the nearby placed VM. The VM was shaped and evacuated with three safety belts closed.

For stabilisation on an LSB the LSB was pressed against the back of the patient and then rotated back with the patient lying on it. SpeedBlocks™ had to be attached and three backboard safety belts closed.

All stabilisation manoeuvres were video-documented with a high-definition digital camera (Everio GZ-HD30, JVC Kenwood Deutschland GmbH, Bad Vilbel, Germany). The camera was equipped with an integrated timer that measured in hundredths of a second.

Before the timer was started, all the equipment was positioned in the same manner in the simulated scene as in the realistic scene. The timer was started as soon as the team leader gave the command to start the stabilisation manoeuvre. The timer was stopped at the moment when the fully immobilized person was lifted up from the ground.

Initially, the time needed for stabilisation on either a VM or an LSB was investigated under ideal conditions. Under ideal conditions, the stabilisation procedure was performed in a training room with the participant lying on a completely level ground (concrete or carpeted floor). Subsequently, stabilisation was performed under realistic conditions in an outdoor environment (level farmland, uneven lawn).

### Statistics

The chi-square test used to compare categorical parameters (qualification, sex) between the LSB and VM groups, and the T-test was used for the procedure time and BMI. Quantile-quantile plots were used to ensure a normal distribution before the T-tests were used. To analyse effect of the stabilisation method on the total procedure time, analysis of variance was used. When there was a significant effect of the method, the methods were also compared with respect to the different conditions and the team members’ levels of qualification. These results were adjusted using the Bonferroni method. The level of significance for all tests was set to be 5 % for α. A pre-hoc power analysis has not been performed as this study has been considered as pilot study regarding this topic. The freeware programme R (Version 2.12, www.r-project.org) was used for the tests. For descriptive characteristics and graphs, Statistica software (version 9.1, StatSoft) was used. BMI was included in the analysis as a confounder since the average values of the groups differed significantly (*p* < 0.05).

## Results

Overall, 172 stabilisation manoeuvres were performed. Seventy-eight stabilisations were carried out on a VM; 51 (65.4 %) were performed in ideal conditions, and 27 (34.6 %) were performed in realistic conditions. Ninety-four stabilisations were carried out on an LSB; 60 (63.8 %) were performed in ideal conditions, and 34 (36.2 %) were performed in realistic conditions. The number of stabilisations performed on a VM respectively on an LSB under ideal and realistic conditions was comparable (*p* = 0.83) but differs slightly since a few stabilisation procedures turned out to be not in accordance with the study protocol in the subsequent video analysis and were therefore excluded. The qualifications of the participating medical personnel varied, but the distribution of personnel with each type of qualification was similar between the two stabilisation groups (*p* = 0.99) (Table 1).

**Table 1:** Characteristics of the personnel and volunteers stratified by the stabilisation method performed

Parameter	Stabilisation method		***p***-value
	Long Spine Board (***n***=94)	Vacuum mattress (***n***=78)	
**Conditions** n (%)			
Ideal	60 (63.83 %)	51 (65.38 %)	0.83
Realistic	34 (36.17 %)	27 (34.62 %)	
**Personnel**			
**Qualification** n (%)			
Emergency physician	11 (11.70 %)	10 (12.82 %)	0.99
Medical student	28 (29.79 %)	24 (30.77 %)	
Paramedic in training	20 (21.28 %)	16 (20.51 %)	
Paramedic	4 (4.26 %)	4 (5.13 %)	
Firefighter	31 (32.98 %)	24 (30.77 %)	
**Participants** n (%)			
**Sex**			
Male	60 (65 %)	51 (65 %)	0.70
Female	23 (25 %)	17 (22 %)	
**BMI**	25.1 (95% CI 24.5 – 25.6)	24.1 (95% CI 23.5 – 24.7)	0.02

The descriptive characteristics are expressed as absolute (relative) frequencies or means with 95% confidence interval in brackets

*n* numbers; *BMI* body mass index; *CI* confidence interval

Note: the number of stabilisation procedures does not match the number of participants since some participants volunteered more than once

The BMI of the participants was 25.1 (95 % CI = 24.5–25.6) in the LSB group and 24.1 (95 % CI 23.5–24.7) in the VM group and thereby slightly below the average BMI of the German population, 26.0 kg/m^2^ [16]. The difference between the groups was statistically significant (*p* = 0.02) but not clinically meaningful (Table 1).

**Fig. 6:**
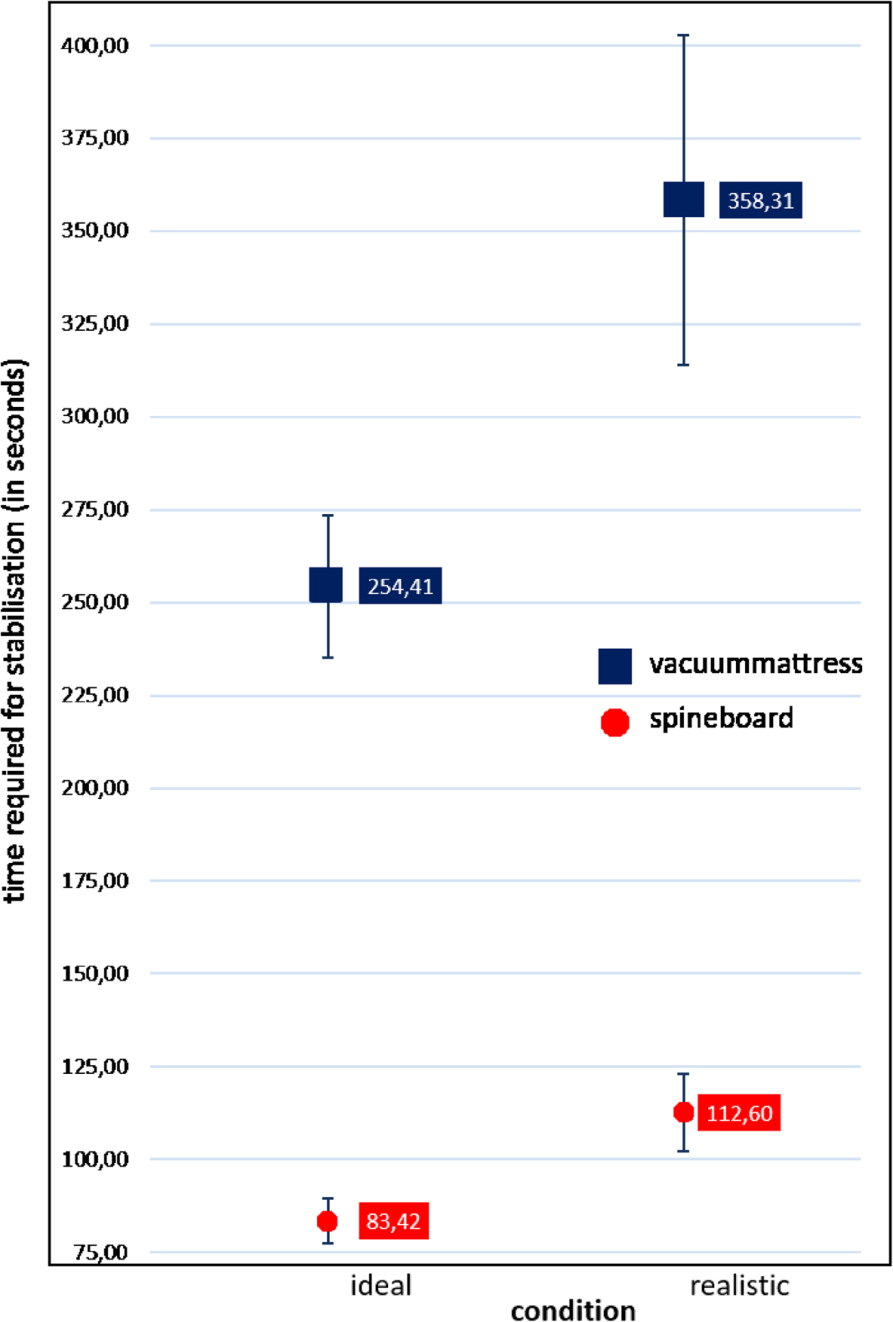
Total treatment time (seconds) versus treatment condition, separately for treatment methods. The figure shows 95 % confidence intervals

Overall, stabilisation on a VM took significantly longer than did stabilisation on an LSB (289.5 s [95 % CI 267.8–311.2 s] vs. 94,0 s [95 % CI 88.1–99.9 s], *p* < 0.01) (Table 2). This finding was observed under ideal (254.4 s [95 % CI 235.6–273.2 s] vs. 83.4 s [95 % CI 77.5–89.3 s], *p* < 0.01) as well as realistic (358.3 s [95 % CI = 316.0–400.6 s] vs. 112.6 s [95 % CI102.6–122.6 s], *p* < 0.01) conditions (Fig. 6), but the difference was even greater under realistic conditions (Δt_real_ 245.7 sec vs. Δt_ideal_ 171.0) (Table 2). The difference between the ideal and realistic conditions in the time required for stabilisation was Δt 103.9 s for the VM method, with the realistic condition requiring 41 % more time, and Δt 29.2 s for the LSB method, with the realistic condition taking 35 % more time. Variation in the time needed for one stabilisation method did not depend on the qualification of the personnel (Table 3).

**Table 2:** Times needed for the stabilisation procedures stratified by the condition

Parameter	Stabilisation method		***p***-value
	Spine board (***n***=94)	Vacuum mattress (***n***=78)	
Total time	94.0 (88.1 – 99.9)	289.5 (267.8 – 311.2)	< 0.01
Total time ideal conditions	83.4 (77.5 – 89.3)	254.4 (235.6 – 273.2)	< 0.01
Total time realistic conditions	112.6 (102.6 – 122.6)	358.3 (316.0 – 400.6)	< 0.01

The mean values with 95% confidence interval in brackets are expressed in seconds

*n* numbers

**Table 3:** Time needed for the stabilisation procedures stratified by qualification of the personnel

Qualification	Spine board	Vacuum mattress	p-value (adjusted)
Emergency physician	90.0 (95% CI 71.3 – 108.8)	295.4 (95% CI 260.6 – 330.1)	< 0.01
Medical student	89.0 (95% CI 81.6 – 96.5)	258.0 (95% CI 236.5 – 279.5)	< 0.01
Paramedic in training	82.9 (95% CI 71.5 – 94.2)	192.5 (95% CI 167.8 – 217.2)	< 0.01
Paramedic	82.3 (95% CI 70.5 – 94.1)	286.1 (95% CI 247.6 – 324.7)	< 0.01
Firefighter	108.8 (95% CI 97.0 – 120.5)	336.9 (95% CI 282.2 – 391.6)	< 0.01

The mean values with 95% confidence interval in brackets are expressed in seconds

## Discussion

In healthy volunteers, LSB stabilisation took an average of 94.0 s (95 % CI 88.1–99.9 s), while VM stabilisation took an average of 289.5 s (95 % CI 267.8–311.2 s) (p < 0.01). With regard to all the trials performed in this study, VM stabilisation took approximately 195.5 s longer than did LSB stabilisation. When realistic conditions were given – which is most likely for severely traumatized patients who might be in extremis – the time required was even longer. Under those circumstances, LSB stabilisation was possible in less than two minutes, while almost six minutes were required for VM stabilisation. One of the contributing factors for more time expended for VM stabilisation were difficulties to proper assemble and separate halves of the sccop stretcher, especially if these parts were put under tension.

It is noteworthy that the variation in the time needed for one stabilisation method did not depend on the qualification of the personnel. Both medical students and paramedics in training have very limited experience in performing stabilisation but required almost the same amount of time as did the paramedics with professional experience. Most likely, this finding indicates that both methods are easy to learn and apply.

It must be realized that these times are applicable only when all necessary equipment is already on site. Thus, it must be taken into account that one helper alone is able to carry all the equipment necessary for LSB stabilisation, while at least two persons are needed to carry a scoop stretcher, a vacuum mattress and a suction pump to the patient, which might also increase the on-scene time.

Another advantage of the LSB is that individuals with injuries can be carried from the site of injury to an ambulance with just two persons, as the LSB offers enough stability. In contrast, a VM is not stable enough, so more than two persons are required for the transport of a patient. For this reason in reality a patient will be most likely be lifted up with a scoop stretcher and then put down on a VM which will be laid out ready on an ambulance gurney. Anyhow this will not influence the results of this study as we measured the time interval until a patient would be ready for transport being stabilized and secured on an evacuated VM, no matter if lying on the ground or in an ambulance.

While this study focussed on the time need for different stabilisation methods it has to be considered that recent guidelines attribute less importance to stabilisation procedures in trauma patients and even raise concern about potential harm of these procedures [3, 5]. The Danish guideline on spinal stabilisation makes a strong recommendation against spinal stabilisation of patients with isolated penetrating trauma and a weak recommendation against a LSB for ABCDE-stable patients as well as a weak recommendation for the use of VM for patient transportation [5]. The Norwegian guideline on spinal stabilisation recommends a selective approach to spinal stabilisation and recommends a strategy of minimal handling [3]. Irrespective of that both guidelines clearly recommend minimal spinal stabilisation in patients with critical ABCD-problems [5] respectively time-critical threat to life [3]. Matching to these recommendations this study demonstrated that a significant amount of time can be saved by using an LSB if SS is indicated and if rapid action is essential in ABCDE-unstable trauma patients who are in extremis. When the target on-scene time is less than ten minutes [9] in particular, the four minutes that can be saved only by choosing a certain stabilisation method can save more than 40 % of the recommended on-scene time. Considering the average on-scene time in Germany is 32 minutes [17], a reduction by 4 minutes is equivalent to 12.5 % of the whole on-scene time. Nevertheless a shorter on scene time per se may not be equated with better outcome [10].

Even though the liberal use of the LSB has been criticized [18] and should be restricted to the prehospital phase of trauma management [19], the LSB is still used for the transport of patients. It should however be realised that the log roll menoeuvre, which most likely is necessary to transfer the patient on an LSB, may cause undue spinal movement [20]. Furthermore patients who are transported on an LSB may develop discomfort, pain and pressure ulcers and that the efficacy according lateral movement is questionable [5, 21, 22]. If used for transport a patient should be moved off an LSB at the earliest possible stage after he arrived in the trauma bay. Therefore risks and benefits for the transport on an LSB must be balanced. If the LSB is used in time-sensitive situations only, such as those requiring an as short as possible prehospital phase these potential risks appear acceptable.

### Limitations of the study

The study was performed with healthy volunteers rather than injured patients, which might influence the results. Nevertheless, we believe that the relative differences between the investigated methods are most likely similar in injured patients.

The quality of stabilisation, e.g., in terms of support of the lumbar spine, was not a part of this investigation. Although this aspect may favour the use of a VM, we believe that it is secondary to the on-site time in critically injured patients.

The sample sizes of the groups were not equal but did not significantly differ since a few stabilisation procedures were not carried out exactly in accordance with the study protocol and were therefore excluded.

## Conclusions

Spinal stabilisation for trauma patients is significantly more time consuming on a vacuum mattress than on a long spine board. Considering that the prehospital time of EMS should not exceed 60 minutes and the on-scene time should not exceed 30 minutes or even 10 minutes if the patient is in extremis, based on our results, spinal stabilisation on a vacuum mattress may consume more than 20 % of the recommended on-scene time. In contrast, stabilisation on a spine board requires only one third of the time required for that on a vacuum mattress.

We conclude that a long spine board may be feasible for spinal stabilisation for critical trauma patients with timesensitive life threatening ABCDE-problems to ensure the shortest possible on-scene time for prehospital trauma treatment, not least if a patient has to be rescued from an open or inaccessible terrain, especially that with uneven overgrown land.

## Data Availability

The datasets of the study are available from the corresponding author on request.

## Abbreviations

BMI: body mass index
EMS: emergency medical services
LSB: long spinal board
SS: spinal stabilisation
VM: vacuum mattress
